# Various painful oral adverse reactions following COVID-19 vaccination: a case series

**DOI:** 10.1186/s12903-022-02100-w

**Published:** 2022-03-08

**Authors:** Youngwoo Chun, Jihee Jang, Jung Hwan Jo, Ji Woon Park

**Affiliations:** 1 Department of Oral Medicine, Seoul National University Dental Hospital, 101, Daehak-ro, Jongno-gu, Seoul, 03080 Republic of Korea; 2 Department of Oral Medicine and Oral Diagnosis, School of Dentistry, Seoul National University, 101 Daehak-ro, Jong-gu, Seoul, 03080 Republic of Korea; 3 Dental Research Institute, Seoul National University, 101 Daehak-ro, Jongno-gu, Seoul, 03080 Republic of Korea

**Keywords:** SARS-CoV-2 vaccination, Oral mucosa, Orofacial pain, Complication, Case report

## Abstract

**Background:**

Adverse events are increasingly being reported with the growing COVID-19 vaccination rate. However, the current literature on orofacial adverse effects following COVID-19 vaccination are severely limited. With the continuation of the global vaccination campaign the incidence of oral adverse effects will inevitably increase.

**Case presentation:**

Clinical characteristics and treatment results of nine patients who complained of pain and discomfort of the oral cavity following SARS-CoV-2 vaccination were analyzed. Swelling and pain of the posterior palatal area, pain on palatal area of the central incisor, pain on the mucosa of the lip and lower gingiva, right preauricular region and right posterior lower gingiva, the buccal mucosa, tongue, and the right lower second molar area were the reported symptoms. Ulceration and swelling of the oral mucosa were found in certain cases. The symptoms were generally mild and responded well to medication within a relatively short period of time.

**Conclusion:**

Oral adverse reactions following COVID-19 vaccination were manageable with treatment. Clinicians should understand the true nature of orofacial adverse reactions following COVID-19 vaccines and guide patients in decision-making.

## Background

Coronavirus disease 2019 (COVID-19) pandemic caused by severe acute respiratory syndrome coronavirus 2 (SARS-CoV-2) started in late 2019 in Wuhan, China [1]. As of August 2021, more than 225 million people have been infected worldwide, and more than 4.6 million people have died of the infection [2]. Various symptoms including dysgeusia, malaise, and respiratory disturbance along with conditions more specific to the oral cavity such as oral ulcerations, stomatitis, petechiae, white oral mucosal lesions, candidiasis, and necrotizing gingivitis have been reported. The exact mechanism behind such oral symptoms is yet to be fully elucidated and some studies are suggesting the possibility of direct SARS-CoV-2 infection of oral keratinocytes in addition to secondary effects of the systemic infection and related treatment [3, 4]. To overcome the spread of the virus several different types of vaccines have been developed in a short period of time. However, there is no consensus on their relative efficacy nor safety. Two RNA-based vaccines (BNT162b2, Pfizer-BioNTech and mRNA-1273, Moderna) and another non-replicating viral vector vaccine (AZD1222, Oxford-AstraZeneca) are being used worldwide [5]. Adverse events are increasingly being reported with the growing vaccination rate regardless of vaccine type. Common symptoms include local pain, redness, swelling, systemic tiredness, headache, muscle pain, fever, and nausea. Regulating authorities are suggesting facial paralysis and swelling as possible orofacial adverse effects of the COVID-19 vaccine, which is assumed to occur rarely [6]. The current literature on orofacial adverse effects following COVID-19 vaccination are limited to a survey-based study, a single case report, and letters to the editor [7–10]. With the continuation of the global vaccination campaign, the incidence of oral adverse effects will inevitably increase. Clinicians should be able to recognize and understand possible oral complications following COVID-19 vaccination for accurate diagnosis and timely treatment. Therefore, this report based on 9 patients who complained of the discomfort of the oral cavity following SARS-CoV-2 vaccination aimed to present clinical manifestations of possible oral adverse reactions and information on provided treatment. The underlying mechanisms of such reactions are also contemplated to offer in-depth knowledge into the relationship between SARS-CoV-2 vaccination and oral adverse reactions.

## Case presentation

This report involved 9 consecutive patients who visited the outpatient clinic of the Department of Oral Medicine from 1st May to 31st June, 2021 with the chief complaint of pain and discomfort of the oral cavity following COVID-19 vaccination. Patients who experienced symptoms more than one month apart from the vaccination were not considered for the report. Information of the included patients is summarized in Table 1. Panoramic radiographs (Orthopantomograph OP 100, Instrumentarium Corporation, Tuusula, Finland) were taken with parameter settings (73 kV, 8 mA) and patient positioning following the standard scanning protocol provided by the manufacturer. Comprehensive laboratory assessments included complete blood cell counts with white blood cell differential, red blood cell indices, erythrocyte sedimentation rate (ESR), and blood chemistry.

**Table 1:** Demographic and clinical information of the cases

Case no	Age	Gender	Type of vaccine	Vaccine dose	Chief complaint	Onset*	Clinical finding
1	79	M	BNT162b2	1st	Swelling and pain of the right posterior palatal area	1 day	Multiple ulcerative lesions with surrounding erythema and slight swelling on right posterior hard palate
2	81	F	BNT162b2	1st	Pain on palatal area of the left central incisor	3 h	Ulceration of the palatal gingiva accompanied by gingival recession and root exposure on the hard palate posterior to both central incisors
3	88	F	BNT162b2	1st	Pain on mucosa of the upper and lower lip and lower gingiva	3 days	Whitish, dried, and cracked lower lip mucosa
4	61	M	AZD1222	1st	Pain on right preauricular region and right posterior lower gingiva	19 days	No specific findings
5	60	F	AZD1222	1st	Pain on right buccal mucosa	1 day	Erythema with whitish lesion on right buccal mucosa
6	66	F	AZD1222	1st	Pain on right border of tongue	3 days	No specific findings
7	68	F	AZD1222	1st	Pain on the right lower second molar area	1 day	No specific findings
8	72	F	AZD1222	1st	Pain on both buccal mucosae	1 day	Erythema with whitish lesion on right buccal mucosa and lower vestibule, mild erythema on left buccal mucosa
9	85	F	BNT162b2	2nd	Pain of tongue	3 days	Fissured tongue with mild tongue coating

F, female; M, male

^*^Time elapsed from vaccination to symptom onset based on patient report

The study was conducted according to the guidelines of the Declaration of Helsinki, and approved by the institutional review board of the hospital (protocol code #ERI 21015, date of approval 6 July 2021). Each patient gave informed consent. The IRB authorized exemption of gaining additional informed consent. The report was described following the guidelines of the Strengthening the Reporting of Observational Studies in Epidemiology (STROBE) statement [11].

### Case 1

**Fig. 1:**
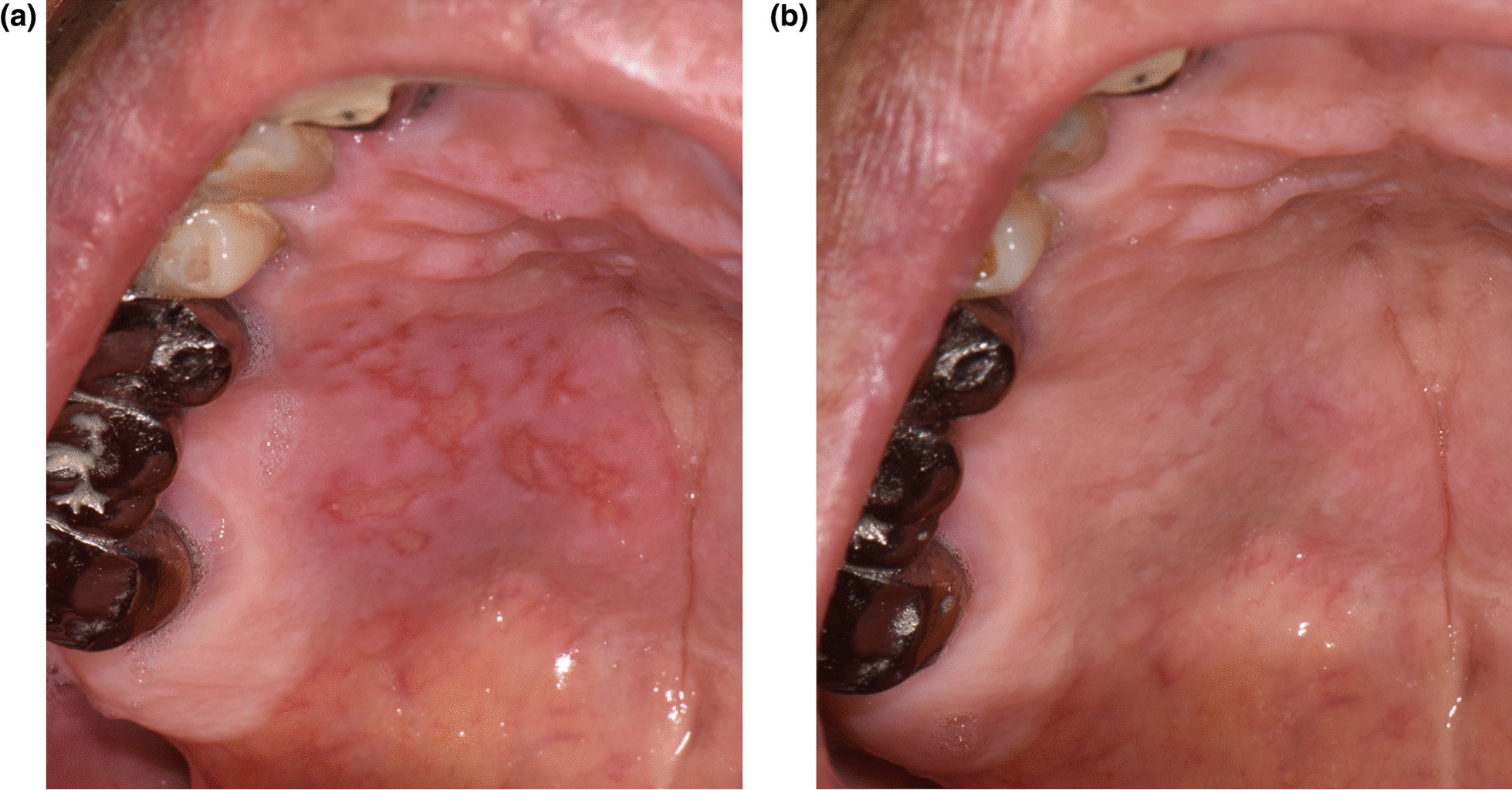
Multiple ulcerative lesions with surrounding erythema and swelling of the right posterior hard palate area before treatment (**A**) and re-epithelialization after 2-weeks of medication (**B**)

A 79-year-old male reported swelling and pain of the right posterior palatal area. The symptoms had occurred a day after his first BNT162b2 vaccination. Past medical history included hypertension and prostatic disease. There was no systemic fever nor any significant findings related to the chief complaint on panoramic radiography. Multiple ulcerative lesions with surrounding erythema and swelling were observed at the right posterior hard palate area (Fig. 1A). The patient reported that symptoms had gradually decreased over the past 5 days without treatment. The initial diagnosis was primary herpetic gingivostomatitis. Dexamethasone solution (0.1%, apply 3 times a day for 15 min a time) was prescribed to suppress mucosal inflammation along with nystatin syrup (100,000 U/ml, apply 3 times a day for 15 min a time) to avoid opportunistic infection. Acyclovir ointment (50 mg/g, 5 times a day) was also prescribed. All medications were given as topical agents. At the 2-week follow up visit, the patient reported almost no discomfort with medication. The ulcerative lesions showed re-epithelialization (Fig. 1B).

### Case 2

An 81-year-old female reported pain on the palatal area of the left central incisor. The symptoms had occurred 3 h after her first BNT162b2 vaccination. The patient also complained of a burning sensation throughout the palate and pain of the tongue. Past medical history included hypertension, regular aspirin intake, and cerebral infarction that occurred 7 years ago. Before the first visit to our clinic, the patient was diagnosed with gingival inflammation at a local dental clinic, and scaling was performed. When the symptoms persisted, the patient visited an otolaryngologist and methylprednisolone (4 mg, 2 times a day for 5 days), acetaminophen (650 mg, 3 times a day for 5 days), trimebutine maleate (150 mg, 3 times a day for 5 days), and benzydamine solution (3 times a day) were prescribed for topical use. The symptoms did not subside after a week of medication, so dexamethasone ointment (1 mg/g), neomycin sulfate (3.5 mg/g), and polymyxin B (6000 IU/g) were additionally prescribed but were not effective. Ulceration of the palatal gingiva accompanied by gingival recession and root exposure was observed on the hard palate posterior to both central incisors. The surrounding gingiva was erythematous and swollen. The tongue was fissured and had tongue coating on the dorsum. There were no pathologic findings related to the chief complaint on standard panoramic radiography. The initial diagnosis was necrotizing gingivitis. Dexamethasone solution (0.1%, apply 3 times a day for 15 min a time), nystatin syrup (100,000 U/ml, apply 3 times a day for 15 min a time), and chlorhexidine gargle (0.1%, apply 2 times a day for 1 min a time) was prescribed as topical agents. At the 1-week follow up visit, the patient reported significant relief of symptoms including tongue pain. The ulcerative lesion showed partial re-epithelialization and gingival regeneration. However, the palatal gingiva still showed slight erythema and swelling. Dexamethasone ointment (1 mg/g, apply 3–4 times a day) was prescribed. On the telephone consultation which took place after 2 months of medication, the patient reported further relief of symptoms with almost no discomfort.

### Case 3

**Fig. 2:**
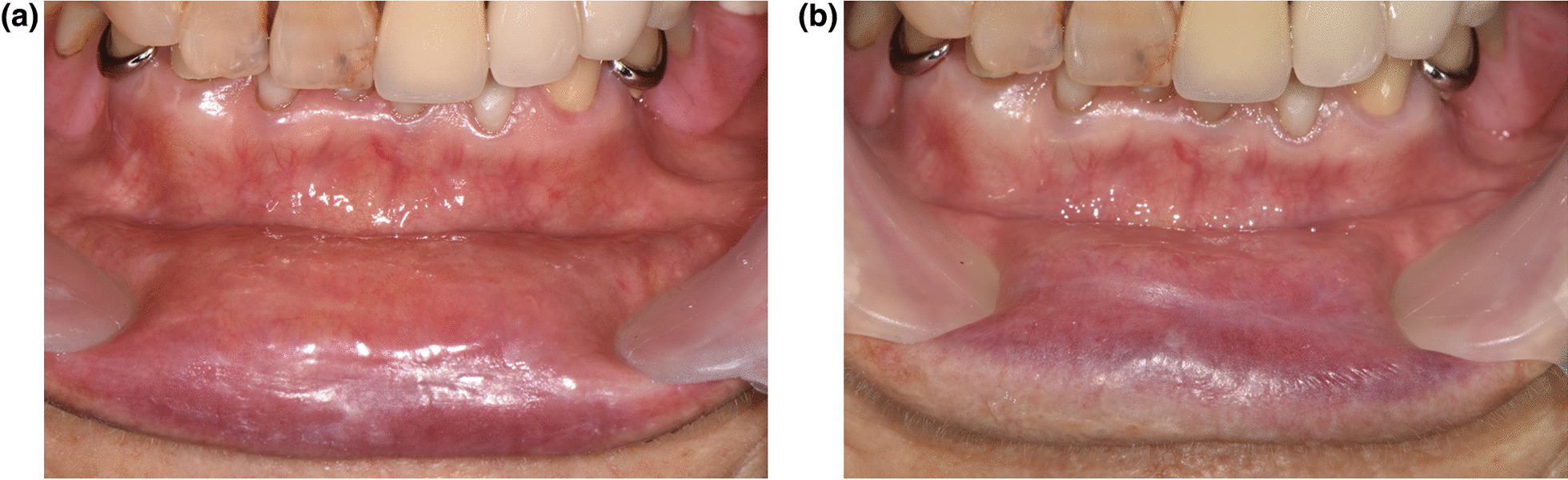
Cracked and dry lower lip before treatment (**A**) and decrease of clinical signs at 1-month follow up (**B**)

An 88-year-old female reported pain on the mucosa of the upper and lower lip and lower gingiva. The symptoms had occurred about 3 days after her first BNT162b2 vaccination. Past medical history included hypertension, hyperlipidemia, recurrent cystitis, and cerebral infarction 5 years ago. Before the first visit to our clinic, the patient had scaling of the lower anterior teeth which resulted in a slight improvement of the gingival discomfort. The patient described the quality of pain as sharp at a level of numerical rating scale (NRS) 8 when provoked and aggravating factors included contact on the lip area and eating spicy or salty food. The pain lasted approximately 10 min per episode. The lower lip was dry and cracked with erythema (Fig. 2A). There were no other significant findings on inspection of the orofacial area. Salivary flow rates were 0.58 ml/min (unstimulated) and 1.38 ml/min (stimulated). There were no significant findings related to the chief complaint on panoramic radiography and psychological evaluation results with Symptom Check List-90-R were within normal limits. Sodium hyaluronate solution (0.25 mg/g, twice a day) and dexamethasone ointment (1 mg/g, three times a day) was prescribed. At 1-month follow up, the discomfort level had subsided from NRS 8 to 6 and clinical signs showed a decrease on examination (Fig. 2B).

### Case 4

A 61-year-old male reported pain in the right preauricular and posterior lower gingival area. The symptoms had occurred 19 days after his first AZD1222 vaccination. Past medical history included hypertension. The quality of pain was electrical and the intensity was NRS 8. The pain occurred only when eating hot food and lasted for 30 s per episode. There were no specific findings related to the chief complaint on panoramic radiography and clinical examination. The patient was provisionally diagnosed with herpes zoster and trigeminal neuropathy. Mecobalamin tablet (1000 μg/day, 7 days), ibuprofen/arginine tablet (400/370 mg, 7 days), acyclovir tablet (800 mg/day, 7 days), and carbamazepine tablet (100 mg/day, 7 days). On a telephone consultation, which took place after 12 days of medication, the patient reported symptom resolution.

### Case 5

A 60-year-old female reported pain on the right buccal mucosa. The symptom had occurred a day after the first AZD1222 vaccination. Past medical history included osteoporosis. The patient was diagnosed with lichen planus of the right buccal mucosa 6 years ago which was totally resolved with dexamethasone (0.1%) and nystatin solution (100,000 U/ml) before. Reported pain intensity was 7 on a 0 to 10 NRS. On clinical examination, the right buccal mucosa showed erythema with white striae. Dexamethasone gargle (0.1%) was prescribed to use 3 times a day for 15 min each time. On the next telephone consultation which took place after a month of medication, the patient reported that the symptom subsided and NRS decreased to 3.

### Case 6

A 66-year-old female reported pain on the right lateral border of the tongue. The symptom had occurred a day after her first AZD1222 vaccination. Past medical history included hypertension, osteoporosis, hyperthyroidism, and headache. Before the first visit to our clinic, chlorhexidine gluconate solution (0.12%) was prescribed, which only exacerbated the symptoms. At another local dental clinic, the patient was diagnosed with neuralgia and clonazepam (0.5 mg/day) and gabapentin (200 mg/day) were prescribed but were not effective. The quality of pain was throbbing and lasted throughout the day. Also, the patient complained of a burning sensation when food or toothpaste contacted the area. Pain intensity was normally NRS 3–7 (flare-up). Panoramic radiography, hematological, and clinical examination revealed nonspecific findings related to the chief complaint. Salivary flow rates were 0.09 ml/min (unstimulated) and 1.40 ml/min (stimulated). The culture test result for Candida infection was negative. The patient was diagnosed with burning mouth syndrome and prescribed clonazepam (0.5 mg/day) and gabapentin (900 mg/day). At 2-week follow up, the pain was reduced to NRS 1–5 (flare-up). The medication regimen was modified to clonazepam (0.5 mg/day and 3 mg topically usage of 1 mg/5 min/time, 3 times a day) and pregabalin (150 mg/day) to reduce the dizziness caused by gabapentin.

### Case 7

A 68-year-old female reported aggravation of pre-existing pain on the right lower second molar. The symptoms had occurred a day after her first AZD1222 vaccination. Past medical history included hypertension, type 1 diabetes mellitus, osteoarthritis, rhinitis, and gastritis. The patient had been diagnosed with neuropathic pain earlier based on the fact that there were no specific findings related to her initial chief complaint on panoramic radiography and clinical examination. The patient previously reported that pain intensity had decreased from NRS 8 to 3 with nortriptyline 10 mg/day. The pain increased following vaccination from NRS 3 to 5. Lidocaine gargle (2%) for topical use and nortriptyline (10 mg/day) was prescribed. At 1-month follow up, the pain was further reduced to NRS 1.

### Case 8

A 72-year-old woman reported pain on both buccal mucosae. The symptom had occurred a day after the first AZD1222 vaccination. Past medical history included diabetes mellitus. The patient was previously diagnosed with oral lichen planus 9 years ago and reported that symptoms were well managed with dexamethasone gargle (0.1%, 1 time/day) and prednisolone (10 mg/day). She reported that symptom aggravation was significant after the vaccination. On clinical examination, the right buccal mucosa and lower buccal vestibule showed erythema with white striae, and the left buccal mucosa showed mild erythema. Dexamethasone gargle (0.1%, 3 times/day) and chlorhexidine (0.1%, 3 times/day) was prescribed. On the next telephone consultation which took place after a month of medication, the patient reported that the symptom had subsided a day after using the medication and there had been no discomfort since.

### Case 9

An 85-year-old female complained of a significant increase in tongue pain that had occurred three days after the second BNT162b2 vaccination. Past medical history included hypertension, osteoporosis, hyperlipidemia, and stroke. The patient had been diagnosed with oral candidiasis and burning mouth syndrome 31 months ago and experienced a decrease in discomfort to a level of no disturbance in daily activities with topical clonazepam (0.5 mg/day for 5 min) and fluconazole syrup (10 mg/ml, 5 cc for 10 min once a day). The patient reported that the first vaccination had not affected symptoms. A fissured and mildly coated tongue was observed on intraoral examination. Symptoms subsided as the patient continually followed the previous medication regimen. On the telephone consultation which took place after 42 days of medication, the patient reported symptom decrease and canceled the next visit.

## Discussion

This is the first case series to report adverse events related to pain occurring in the oral cavity following SARS-CoV-2 vaccination found at a dental clinic. Oral mucositis, mucosal ulceration, and aggravation of preexisting neuropathic pain were the main symptoms that could be observed. Such findings are in line with previous survey studies based on structured questionnaires showing oral blisters, ulcers, and vesicles, along with paresthesia as the most frequently reported orofacial side effects [12–14].

Due to the current limitation in related literature, it is difficult to explain the exact mechanism underlying such symptoms following COVID-19 vaccination and due to the nature of this report the presented symptoms may have been caused by other reasons unverified. Temporality is the major Hill’s criteria that is met in this case series along with indirect plausibility to infer causality however, other criteria including strength, consistency, and specificity of the association could not be verified [15]. Also, there is the possibility that the oral mucositis and ulceration had been caused by an infection that occurred with vaccination. In that case, the area and characteristics of the lesion could be related to the key protein, angiotensin I converting enzyme 2 which is involved in host cell entry. This functional receptor can be found in many parts of the human body and is highly expressed on epithelial cells of the tongue and salivary glands [10, 16]. However, vaccines based on adenovirus and mRNA are unlikely to directly infect host cells and routes of transmission through the oral mucosa are yet to be confirmed. Another point to consider is the incubation period of COVID-19 which has been reported to be 5.1 days with more than 95% of the patients showing symptoms within 11.5 days from infection [17]. The onset of symptoms generally occurred within 3 days of vaccination with our cases, limiting the possibility of a direct infection by the vaccine itself. Hypersensitivity reactions to certain vaccine components could also cause oral mucositis and ulcers. An immunoglobulin (Ig) E response could be elicited by proteins present in the vaccine that are non-target antigens. Such proteins are present in the cell culture medium used to cultivate the virus during vaccine production. However, this is also unlikely since a typical type 1 hypersensitivity reaction occurs within 30 min but still, a more delayed type III Ig G based immune reaction could also take place [18]. Adjuvants within vaccines may cause specific autoimmune adverse symptoms known as autoimmune/inflammatory syndrome induced by adjuvant (ASIA) which is a result of molecular mimicry resulting from the significant similarity between certain vaccine elements and human proteins. The similarity may trigger immune cross-reactivity. Also, autoreactive B and T cells may be activated by vaccine antigens expressed on keratinocytes, leading to a CD8+ T-lymphocyte response against epidermal cells [19]. AZD1222 vaccine has been shown to produce a T-cell-specific response [20]. Such a mechanism could explain the aggravation of oral lichen planus observed in 2 cases of this report and a previous case report on Stevens-Johnson syndrome which are both T-cell-mediated inflammatory diseases [8, 21]. One interesting finding with our cases that has not been previously reported in the literature is the significant increase in pain levels of preexisting pain disorders of the tongue and oral mucosa that had been well controlled with medication. The major role of the immune system in neuropathic pain has been repeatedly reported and the activation of immune cells by vaccination could have caused the acute flare-ups observed in this study through direct communication between nociceptive neurons and immune cells [22].

All patients of this report were over 60 years of age (mean 73.3 ± 10.3 years) partly due to the national vaccination schedule. This result is distinguished from previous studies showing a higher rate of vaccine adverse events in younger people. Both local and systemic adverse reactions with the BNT162b2 vaccine were more frequent in the 18 to 55 years age group compared to the > 55 years group (82.8% vs 70.6% and 88.7% vs 79.7%, respectively) [23]. This may be due to a higher S-protein-targeted neutralizing antibody response reported in older adults compared to younger adults or could be a more specific characteristic of oral mucosal side-effects [24]. Adverse reactions of the oral cavity may occur more frequently in immune dysregulated individuals or those with preexisting diseases [22]. The investigation should be expanded to include younger populations to elucidate the true effect of age on the occurrence of adverse effects from vaccination. Seven of the nine patients were female, suggesting a difference in susceptibility to adverse effects according to sex. This is in line with results from other vaccines. Women had four times more immediate hypersensitivity reactions than men after monovalent 2009 pandemic influenza A (H1N1) vaccines [25]. Previous literature showed that when injected with H1N1 vaccine, females aged 18–45 developed higher interleukin-6 and antibody responses compared to adult males or females over 65, with female antibody responses showing a positive association with estradiol concentration [26]. Further prospective investigations involving diverse study groups of different ages and systemic conditions including healthy populations are needed to establish the direct influence of various factors on adverse reactions due to COVID-19 vaccination.

The mere possibility of adverse effects can cause vaccine hesitancy, which is referred to as a delay or refusal of vaccines despite their availability. The rate of COVID-19 vaccine hesitancy can be as high as 69.6% leading to a delay in the accomplishment of herd immunity [27]. However, adverse reactions of this study were generally mild and all patients experienced significant symptom relief with medication within a relatively short period of time showing benefits from vaccination far outweigh its risks. Previous literature has suggested the incidence rate of adverse orofacial reactions following various types of vaccinations as low as 1 in 1000 people and this number should not differ greatly with COVID-19 vaccination. Also, the reported symptoms are not much different from those related to polio and diphtheria vaccination which include gum bleeding, sores, white spots, ulcers, and halitosis [6]. Since concern about side effects is the most common reason for hesitancy, clinicians should understand the true nature of orofacial adverse reactions to COVID-19 vaccines and its favorable response to treatment to be able to guide patients in decision-making related to the COVID-19 vaccine [28].

## Conclusion

This case series suggest oral mucositis, ulceration, and neuropathic pain triggering as possible orofacial adverse reactions following COVID-19 vaccination. The symptoms were generally mild and responded well to conventional treatment. Clinicians should be able to understand the clinical manifestations of orofacial adverse reactions with COVID-19 vaccination and guide patients to lower vaccine hesitancy.

## Data Availability

The datasets used during the current study are available from the corresponding author on reasonable request.

## Abbreviations

ASIA: Autoimmune/inflammatory syndrome induced by adjuvant
COVID-19: Coronavirus disease 2019
ESR: Erythrocyte sedimentation rate
NRS: Numerical rating scale
SARS-CoV-2: Severe acute respiratory syndrome coronavirus 2
